# Magnet Beads Impacted in the Appendix of a Child: A Case Report and Review of the Literature

**DOI:** 10.7759/cureus.9777

**Published:** 2020-08-16

**Authors:** Khaled Nazzal, Osama Nazzal, Alya Ahmed, Husain Alaradi, Saeed Alhindi

**Affiliations:** 1 Surgery, Salmaniya Medical Complex, Manama, BHR; 2 Surgery, Arabian Gulf University, Manama, BHR; 3 Internal Medicine, Salmaniya Medical Complex, Manama, BHR; 4 Pediatric Surgery, Salmaniya Medical Complex, Manama, BHR

**Keywords:** foreign body ingestion, impacted in the appendix, magnet beads, pediatric case

## Abstract

Foreign body ingestion is a commonly encountered problem in the pediatric population, which can be a source of severe distress to parents and caregivers. Certain foreign bodies such as magnets, bones, and button batteries can be particularly dangerous and lead to some serious complications like gastrointestinal obstruction, perforation, or bleeding depending on the nature of the foreign body, the location of impaction, and the period since ingestion. In this report, we discuss a case of a 23-month-old girl who ingested multiple magnets that got trapped within the appendix resulting in continuous vomiting.

## Introduction

Children's innate love to experience their surroundings can be frequently manifested as the ingestion of foreign bodies. From the variety of foreign bodies that could be ingested by children, magnets are considered to be specifically dangerous if >1 magnet is ingested due to their ability to attract and pull each other across bowel layers, leading to pressure necrosis, perforation that could lead to sepsis, or even death [1,2]. Management of such cases varies according to multiple factors, such as clinical presentation, the number of ingested magnets, location of the magnets, and ensuing complications [3]. Follow-up with serial imaging can be carried out as a form of initial management in stable patients. However, in unstable patients, clinical deterioration, and failure of magnets to progress through the gastrointestinal tract with failure of retrieval endoscopically, surgical intervention is indicated [2,3].

## Case presentation

A 23-month-old baby girl was brought to the accident and emergency department by her parents with a complaint of non-projectile vomiting of two days' duration. Before the presentation, she had vomited up to 30 times per day. The vomitus initially consisted of gastric secretions and milk, but later on, she vomited coffee-ground like material. Associated symptoms included lethargy, decreased oral intake, and activity. However, there was no history of melena, constipation, or diarrhea. On clinical examination, she was conscious, but she looked tired and irritable. The vital signs showed mild tachycardia with a heart rate of 135, and her blood pressure and temperature were within normal limits. Given the history of the patient, signs of dehydration were tested for and indicated a state of moderate dehydration with a capillary refill time of two seconds and slightly dry mucus membranes and skin. Upon examining the abdomen, it was soft, lax, not distended, without tenderness, and with no palpable organomegaly.

**Figure 1:**
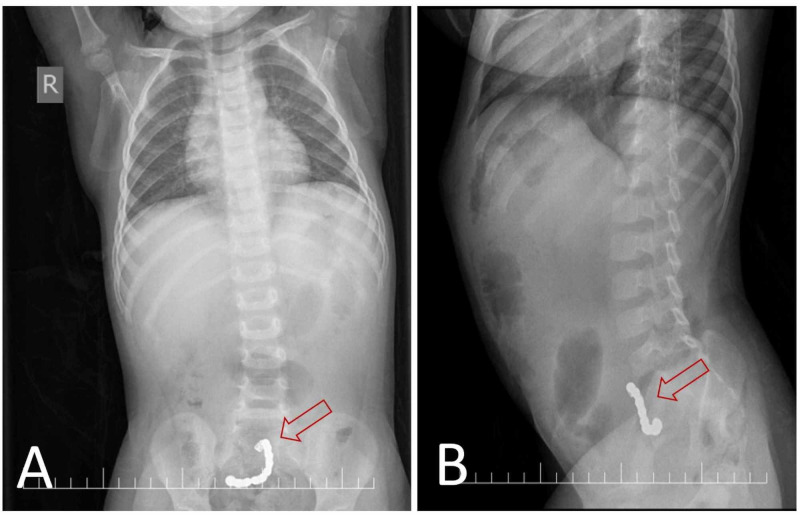
Abdominal X-ray. (A) Postero-anterior abdominal X-ray image. (B) Lateral abdominal X-ray image. Images show a chain of 11 beads extending from the hypogastric region to the right iliac fossa.

Preliminary blood investigations were ordered, including a full blood count, which showed leukocytosis (11.81 × 10^9^/L) with neutrophilia (79.5%) and high platelet count (638 × 10^9^/L). The coagulation profile, renal function test, and routine electrolytes were unremarkable. Given the history of continuous vomiting, an abdominal X-ray was ordered and showed a chain of 11 beads extending from the hypogastric region to the right iliac fossa. However, no signs of small bowel obstruction were identified (Figure 1).

**Figure 2:**
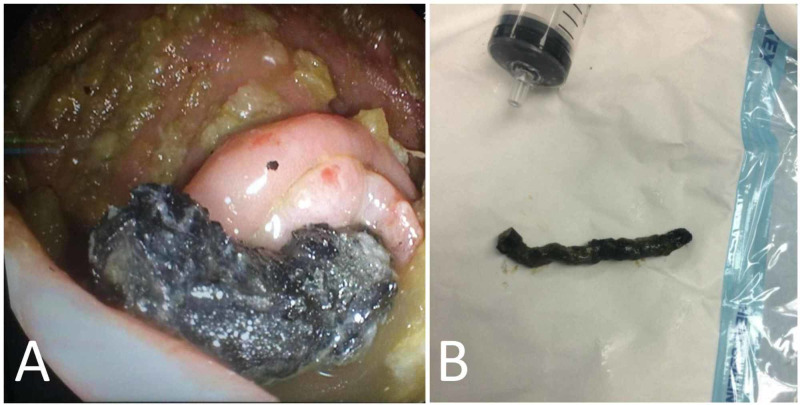
Colonoscopy. (A) Image from colonoscopy revealing a chain of 11 magnets with a hook impacted in the appendix. (B) The 11 magnets chain after being removed by colonoscopy.

The patient was admitted for observation and possible surgical intervention. She was kept on proper fluid replacement and was monitored closely with serial clinical examinations and serial abdominal x-rays, which showed no change in the location of the foreign bodies. She was given laxatives and rectal enemas to encourage bowel emptying and to prepare the bowel for possible surgery. On the second day, the vomiting decreased to four times and she passed stool with no evidence of passing the foreign bodies. On the third day, the patient underwent a colonoscopy that revealed 11 magnets in a chain with a hook, which was impacted in the appendix; they were removed with the help of endoscopic forceps (Figure 2).

The patient was discharged with an oral antibiotic on the next day. She was followed up after two weeks and showed prompt recovery.

## Discussion

Foreign body ingestion is most common among children in the stage of exploratory development between the ages of six months and six years [4]. The most common ingested foreign bodies are coins [1,5]. Other commonly ingested foreign bodies include fish bones, pins, button batteries, magnets, household items, food boluses, and many others. They can get stuck anywhere in the gastrointestinal tract, starting from the proximal esophagus to the colon, manifesting in the form of various signs, symptoms, and complications [3,6].

The ingestion of magnets is a serious health hazard for children that can lead to grave consequences if not managed promptly. Multiple magnets across the gastrointestinal tract can attract each other, thus attracting different segments of the bowel together [3]. This can eventually lead to bowel ischemia, enteroenteric fistulas, bowel perforation, and peritonitis [3,6]. Although ingested foreign bodies can be found anywhere in the gastrointestinal tract, they are rarely lodged within the vermiform appendix. Thin and sharp objects like pins were found to be the most common foreign bodies to be displaced in the appendix, with a higher risk of perforation [7]. A foreign body in the appendix is difficult to come out to the large intestines again; thus, it can lead to appendicitis, bowel obstruction, necrosis, perforation, and peritonitis [8]. Once the foreign body is suspected to be impacted in the appendix, active management options should be taken like endoscopic removal or surgical intervention depending on the patient's state and bowel condition [8].

To the best of our knowledge, this is the first report of magnets being impacted in the appendix. Other foreign bodies encountered in the pediatric population and found in PubMed and Google Scholars were metallic screws, needles, firearm pellets, hazelnut, canine hair, sand and stones, metal coil, seeds, the tip of mercury thermometer, plastic pieces, metal beads, nail, and a small blunt metallic object [7-19]. Upon reviewing the previous case reports, 21 cases were found. The most common foreign bodies encountered were thin and sharp objects like needles and screws. The youngest of the patients was an 11-month-old boy, and the oldest was a 13-year-old boy [9,16]. Although some patients were asymptomatic, most of them were symptomatic with complaints of abdominal pain, vomiting, fever, anorexia, diarrhea, and irritability. The most common presenting symptoms were abdominal pain, vomiting, and fever. These cases showed that the vast majority of the patients were managed surgically in the form of appendectomy or prophylactic appendectomy. On the other side, only one patient was closely observed until he passed the foreign body [15]. Our patient presented with continuous vomiting and decreased activity. We elected to manage her case via a colonoscopic retrieval of the foreign body, which we found to be efficient, minimally invasive, and safe.

## Conclusions

The choice of how to approach a foreign body impacted in the vermiform appendix is selected on a case-to-case basis, depending on the presentation, complications, and expertise. In our case, attempting to retrieve the object via colonoscopy proved to be an efficient and safe option, thus dispensing a definitive surgical intervention. Lastly, we emphasize the importance of protecting children and keeping any possible dangerous objects away from their hands.

## References

[1] Jayachandra S, Eslick GD (2013). A systematic review of paediatric foreign body ingestion: presentation, complications, and management. Int J Pediatr Otorhinolaryngol.

[2] Naji H, Isacson D, Svensson JF, Wester T (2012). Bowel injuries caused by ingestion of multiple magnets in children: a growing hazard. Pediatr Surg Int.

[3] Wang K, Zhang D, Li X (2020). Multicenter investigation of pediatric gastrointestinal tract magnets ingestion in China. BMC Pediatr.

[4] Khorana J, Tantivit Y, Phiuphong C, Pattapong S, Siripan S (2019). Foreign body ingestion in pediatrics: distribution, management and complications. Medicina.

[5] Cheng W, Tam PK (1999). Foreign-body ingestion in children: experience with 1,265 cases. J Pediatr Surg.

[6] Kramer RE, Lerner DG, Lin T (2015). Management of ingested foreign bodies in children: a clinical report of the NASPGHAN Endoscopy Committee. J Pediatric Gastroenterol Nutr.

[7] Samujh R, Mansoor K, Khan I, Mannan A (2007). 'Screw'-appendicitis. Indian Pediatr.

[8] Lee M, Kim SC (2017). Appendiceal foreign body in an infant. Medicine.

[9] Kumar R, Bawa M, Ragavan M (2010). Ingested metallic screw causing appendicitis in an infant: the metallic ‘screw appendicitis’. Indian J Pediatr.

[10] Sarkar RR, Bisht J, Sinha Roy SK (2011). Ingested metallic foreign body lodged in the appendix. J Indian Assoc Pediatr Surg.

[11] Yildiz H, Okay ST, Yildirim E, Beskardesler N (2020). A pin detected by ultrasonography within the normal appendix: prior to surgery, an impressive use of ultrasonography to localize an ingested foreign body exactly [Epub ahead of print]. J Ultrasound.

[12] Llullaku SS, Hyseni NSh, Kelmendi BZ, Jashari HJ, Hasani AS (2010). A pin in appendix within Amyand's hernia in a six-years-old boy: case report and review of literature. World J Emerg Surg.

[13] Hartin CW Jr, Lau ST, Caty MG (2008). Metallic foreign body in the appendix of 3-year-old boy. J Pediatr Surg.

[14] Zampieri N, Zuin V, Ottolenghi A, Camoglio FS (2008). Recurrent abdominal pain due to buckshots in the appendix. Acta Pædiatr.

[15] Volovelsky O, Gross E, Shteyer E (2013). Appendicular foreign body: patience needed. J Pediatr Surg.

[16] Hulme P (2010). Foreign body causing perforation of the appendix in an African boy. Pan Afr Med J.

[17] Lambropoulos V, Fotoulaki M, Kepertis C, Neofytou A, Spyridakis I (2016). An unusual foreign body within the appendix. Med J Malaysia.

[18] Mohammed AA, Ghazi DY, Arif SH (2019). Ingested metallic foreign body impacted in the vermiform appendix presenting as acute appendicitis: Case report. Int J Surg Case Rep.

[19] Brown J, Kidder M, Fabbrini A, deVries J, Robertson J, Chandler N, Wilsey M (2019). Down the rabbit hole-considerations for ingested foreign bodies. Pediatr Gastroenterol Hepatol Nutr.

